# Hypokalemic periodic paralysis, a rare yet critical condition: A case report

**DOI:** 10.3892/mi.2025.220

**Published:** 2025-02-14

**Authors:** Saime Paydas, Mehmet Ali Gergerli, Ahmet Celik

**Affiliations:** 1 Adana Acıbadem Hospital, Adana 01130, Turkey; 2 Mehmet Akif Inan Training and Research Hospital, Republic of Turkey Ministry of Health, Şanlıurfa 63040, Turkey

**Keywords:** hypokalemia, paralysis, trigger factor, channelopathy, intracellular potassium

## Abstract

Hypokalemic periodic paralysis (HPP) is a rare disease. Due to channelopathy caused by mutations in skeletal muscle ion channels, episodes of sudden flaccid muscle weakness and hypokalemia develop as a result of various trigger factors. The present study reports the case of a 25-year-old male patient with HPP admitted with acute onset numbness and paralysis in the extremities accompanying hypokalemia (2.66 mEq/l). The patient became asymptomatic following treatment with a potassium (K) supplement and was diagnosed with HPP. The present study describes this case of HPP in an aim to remind colleagues of the possibility of HPP in hypokalemic patients with muscle weakness and flaccid paralysis.

## Introduction

Hypokalemic periodic paralysis (HPP) is a rare muscle membrane ion-channel disease (1,2). Attacks are related to the sudden onset of muscle flaccidity due to low serum potassium (K) levels and redistribution. The serum K level is normal between attacks. The proximal muscles of the extremities are more commonly affected than the distal muscles, and respiratory, ocular and bulbar involvement can be observed. A trigger factor is required for an attack in patients with HPP (3). Trigger factors include exercise, stress, a carbohydrate-rich diet, the use of β-adrenergic agonists, alcohol consumption, cold weather and increased levels of insulin or epinephrine. The present study reports the case of a 25-year-old male patient with HPP who was admitted to an emergency department with paralysis, weakness and difficulty in walking.

## Case report

### Patient

A 25-year-old male was admitted to the Emergency Department of Mehmet Akif Inan Training and Research Hospital (Şanlıurfa, Turkey) reporting from difficulty in walking, weakness and numbness in the hands and feet. He stated that his symptoms developed after consuming a carbohydrate-rich meal. The patient had no history of medication, alcohol consumption, drugs or chronic disease. His previous medical history included hospital admissions several times in the previous 2 years due to cough and fever, which were treated with dexamethasone and antihistamines. His last dexamethasone treatment was 6 days prior to the occurrence of the muscle weakness and flaccid paralysis. He had no history of headache, visual impairment or loss of consciousness. His family history was negative for signs and symptoms suggesting HPP.

### Physical examination

The patient was oriented and his vital signs were normal. There was no pathological finding apart from for muscle weakness (2/5) in the extremities, and his deep tendon reflexes were weak.

### Blood test results

The blood test results of the patient are presented in Table I. The results of other tests were within normal limits, as follows: HbA1C, 4.30; aspartate aminotransferase, 20 U/l (normal range, 5-40 U/l); alanine transaminase, 43 IU (normal range, 5-41 IU); gamma-glutamyl transferase, 27 IU (normal range, 7-60 IU); bilirubin, 0.76 mg/dl (normal range, 0.2-1.2); magnesium, 2.24 mg/dl (normal range, 1.6-2.6 mg/dl); T3, 3.74 pg/ml (normal range, 1.71-3.71 pg/ml); T4, 1.02 uIU/ml (normal range, 0.7-1.48 uIU/ml); aldosterone/renin (<30 ng/dl:ng/ml/h), 15.36 (March 29, 2024). Urinalysis revealed normal results and an electrocardiogram also yielded normal results. A cerebral computerized tomographic scan and cerebral-cerebellar diffusion magnetic resonance imaging also yielded normal results.

### Patient management

A total of 20 mEq K was infused for hypokalemia and the accompanying paralysis. Muscle weakness gradually decreased and resolved after 6 h and the serum K level increased from 2.66 to 3.99 mEq/l. He had become asymptomatic by this point and was discharged with oral K supplement. In the follow-up period the serum K level was within normal limits in all measurements, except for values of 3.22 mEq/l and 3.32 mEq/l on the 75th and 76th days, respectively. No abnormalities were detected in thyroid function and venous blood gases. In May, treatment with spironolactone (25 mg/day) was commenced for his high normal blood pressure.(120-139/70-89 mmHg in office) and K supplement (kalinor 3x1 tablet/day (one tablet contains 2.170 g potassium citrate, 2.000 g potassium bicarbonate, 2,057 g anhydrous citric acid). Following this attack, in February, 2024 the patient had three further attacks in May, June and July of the same year with hand tremors and spasms, while he was taking a chronic K supplement and spironolactone (25 mg/day) therapy. His attacks resolved with an additional (one tablet kalinor) K supplement at home.

## Discussion

HPP is a channelopathy associated with mutations affecting the sodium and calcium channels (4-6). These mutations disrupt the normal electrical current via voltage domains, leading to there being no stimulation in muscle cell membrane (7). Muscle action does not function and the muscles become flaccid. Arrhythmias, including ventricular tachycardia/fibrillation and atrioventricular block may develop due to hypokalemia and can be life-threatening. In the case described herein, there were no electrocardiographic abnormalities.

In HPP the proximal muscles of the lower and upper extremities are most commonly affected, while the respiratory muscles are rarely involved. Vesical globe has been reported (8). The total bodily amount of K does not change: K passes into the cell during an attack and returns to the plasma afterwards.

In addition to mutations, various trigger factors may initiate attacks. These factors are insulin, a high carbohydrate diet, alkalosis, adrenaline and heavy exercise. These facilitate the entry of K into the cell (3) and COVID-19 infection. In the case presented herein, the triggering factor was a carbohydrate-rich meal. Alkalosis was not detected. His glucose level was not severely high and his blood sugar was 160 mg/dl.

Another trigger factor in the case described herein may have been steroid therapy (9). The patient in the present study had been treated with steroids 6 days prior. In fact, steroid-induced proximal muscle weakness is a common finding related to the loss of K due to the mineralocorticoid effect of steroids. In addition, K passes into the cell due to steroid-induced hyperglycemia and hyperinsulinemia. Insulin increases the activity and number of Na-K ATPase, and K entry into the cell. Insulin also inhibits the inward rectifier K channel (Kir) current, leading to a more depolarized membrane potential. Another effect is that steroids upregulate β2 receptors in the cell membrane and have a stimulating effect on the interaction between catecholamines and β2 receptors in Na-K ATPase (10,11). The patient in the present study did not describe a similar situation as regards previous steroid therapy; however, this possibility cannot be completely excluded.

HPP due to COVID-19 infection has also been reported as a trigger factor (12). There were no signs or symptoms of infection in the patient described herein. His aldosterone, plasma renin activity and catecholamine levels were within normal limits. The levels of serum K (when there was no attack) chloride and blood gas pH levels were also within normal limits. The weakness in the arms and legs felt by the patient improved within 6 h with K supplementation and he was discharged after 12 h of monitoring.

Thyrotoxicosis may also present a similar image, but was not detected in the patient in the present study. High levels of thyroid hormone, catecholamines and insulin contribute to the development of hypokalemia by preventing the excretion of K from the cell (13,14). Since estrogen affects the transfer of K into the cell, thyrotoxic HPP is more frequent and severe in males than in females.

In HPP, flaccid paralysis usually develops when the serum K level is <3 mEq/l. The serum K level in the patient described herein case was 2.66 mEq/l. When the serum K level is <3 mEq/l, the affected muscle fibers paradoxically become continuously depolarized and muscle cannot be electrically stimulated. However, in unaffected normal fibrils, hyperpolarization occurs when the serum K level decreases. Normally, the inward rectifier K channel (Kir) and membrane ATPase maintain a negative resting membrane potential.

HPP is observed in ~1 in 100,000 cases, has an autosomal dominant transmission pattern and sometimes occurs sporadically (9). Flaccid paralysis attacks in HPP begin at an early age (3). The patient in the present study was 25 years of age. His disease may have had a late onset or there may have been a delay in diagnosis. The family members of the patient had no HPP-like symptoms or diagnosis. Family history is critical in diagnosing HPP, although its absence does not exclude such a diagnosis. Conducting a genetic test for HPP mutation can facilitate prognosis and may help in terms of family screening; the importance of these tests for targeted therapy will increase in the future. Currently, however, only a small number of gene locations are tested, and for this reason, almost 1 in 3 patients with a clinical HPP diagnosis has no known mutation.

HPP can be diagnosed through a specialized form of electromyographic (EMG) testing. Compound muscle action potential (long exercise test) measures the amplitude of nerve responses for 40-50 min following a few minutes of exercise. In patients with HPP, the amplitude progressively decreases. Compound muscle action potential is the current standard diagnostic test during an attack or between attacks, or to assess whether the K level is low or normal (2). As regards the patient in the present study, it was not possible to conduct EMG testing. Standard EMG testing can only confirm the diagnosis during an attack. In the patient described herein, the secondary attacks were not observed in hospital; thus, EMG could not be used. In addition, it is dangerous to provoke an attack in order to make a diagnosis. HPP was diagnosed based on the medical history and clinical and laboratory findings.

In patients with HPP, inhalational anesthetics and muscle relaxants, such as succinylcholine can cause malignant hyperthermia during anesthesia, which is a dangerous situation. It is important to inform the patient about this problem.

The frequency of attacks in HPP is variable. They can occur once or twice a week or more, or, alternatively, very rarely. Following the diagnosis, the patient in the present study did not attend the hospital with similar symptoms for 5 months. The patient described extreme fatigue and cramps in his hands and feet in a video-recorded consultation. It is not known what his serum K value was at these symptomatic periods, although he described improvements in symptoms with an additional K supplement at home.

HPP attacks impair the quality of life of patients. In a previous cross-sectional study using the individualized neuromuscular disease questionnaire, it was determined that the quality of life of patients worsened with increasing age. Muscle weakness and accompanying fatigue were the key factors on the quality of life (15).

The limitations of the present report are the lack of specific EMG testing regarding compound muscle action potential, the lack of standard EMG testing during an attack and stimulation tests (which can dangerous), and the lack of a genetic test (which is negative in >30% of patients).

In conclusion, in the present study, hypokalemia was diagnosed in a 25-year-old male who applied to the Emergency Department of Mehmet Akif Inan Training and Research Hospital due to flaccid paralysis weakness and difficulty walking accompanying hypokalemia and the presence of a trigger factor. Following K therapy, his clinical condition and hypokalemia improved in 6 h. The patient was informed about trigger factors and the risk when using inhalational anesthetics. During the follow-up period, while the patient was under K and Aldactone (spironolactone) treatment, mild attacks were described that resolved at home after increasing the oral K dose. In this regard, for patients with muscle weakness and flaccid paralysis during emergency room visits, it is critical to measure the serum K level and to consider HPP as a differential diagnosis.

## Figures and Tables

**Table I:** Laboratory results of the patient during the attack.

Parameter	Concentration
Serum potassium, mEq/l (normal range, 3.5-5.1 mEq/l)	2.66 (04:45 h); 3.99 (10:13 h); 4.08 (10:29 h)
Serum bicarbonate, mmol/l (normal range, 23-28 mmol/l)	21.4 (04:45 h); 21.1 (05:44 h); 20.4 (10:13 h)
Venous blood pH, (normal range, 7.31-741)	7.364 (04:45 h); 7.345 (05:44 h); 7.377 (10:13 h)
White blood cells, cells/µl (normal range, 3.7-10.1 cells/µl)	15.1
Hemoglobin, g/dl (normal range, 12.9-15.9 g/dl)	14.7
Platelets, cells/µl (normal range, 155,000-400,000 cells/µl)	357,000
Glucose, mg/dl (normal range, 74-106 mg/dl)	161
Serum sodium, mEq/l (normal range, 136-145 mEq/l)	141
Blood urea nitrogen, mg/dl (normal range, 10-50 mg/dl)	20
Creatinine, mg/dl (normal range, 0.7-1.2 mg/dl)	0.9
Serum aldosterone, ng/Dl	7.09 (February 19, 2024); 34.42 (March 29, 2024)
Plasma renin activity, ng/ml/h	0.33 (February 19, 2024)
Aldosterone/renin (<30 ng/dl:ng/ml/h)	21.48 (February 19, 2024)
Thyroid stimulating hormone (0.35-4.94 ıUl)	0.992
Catecholamines (<90 pg/ml)	68.03 (March 29, 2024)
Catecholamine metabolites (<500 ng/ml)	171.86 (March 29, 2024)

## Data Availability

The data generated in the present study may be requested from the corresponding author.
